# Disease Activity and Bone Mineral Density of MCP Joints in Patients with Rheumatoid and Psoriatic Arthritis: Is There a Correlation?—A Study in Patients Treated with Methotrexate and an Anti-TNF**α** Agent

**DOI:** 10.1155/2013/708323

**Published:** 2013-12-07

**Authors:** Ilaria Bertoldi, Georgios Filippou, Carlo Alberto Scirè, Valentina Picerno, Valentina di Sabatino, Antonella Adinolfi, Serena Pierguidi, Mauro Galeazzi, Bruno Frediani

**Affiliations:** ^1^Department of Medicine, Surgery and Neurosciences—Rheumatology Department, University of Siena, Policlinico le Scotte, Viale Bracci 16, 53100 Siena, Italy; ^2^Epidemiology Unit, Italian Society for Rheumatology (SIR), Milan, Italy

## Abstract

*Background*. Bone damage in rheumatoid arthritis (RA) and in psoriatic arthritis (PsA) includes an accelerated bone mineral density (BMD) reduction. The objective was to evaluate BMD variations of the metacarpophalangeal joints (MCPs) in patients starting treatment with methotrexate (MTX) or etanercept. *Methods*. Patients affected by RA or PsA with hand joints involvement and with moderate or high disease activity, were enrolled in this study. All patients underwent clinical examination, laboratory exams, and a DXA scan of the most affected hand, as assessed with an ultrasound examination at the baseline, at the time of enrolment and after 1, 3, 6, and 12 months. Patients non-responders to MTX received combination therapy, while patients with no previous treatment initiated MTX. *Results*. 22 patients were enrolled. In both RA and PsA groups, BMD increased independently of the treatment. However, in the patients affected by RA, a slight BMD decrease was observed at the last checkup. Globally, the BMD variations of the MCPs were strongly correlated with the disease activity. At the reduction of DAS28, the scores corresponded an increase of BMD. *Conclusions*. MCPs BMD is inversely correlated to disease activity. BMD increase seems to be correlated with the response to treatment and not with the drug itself.

## 1. Introduction

Bone damage in rheumatoid arthritis (RA) and in psoriatic arthritis (PsA) includes joint damage and accelerated bone mineral density (BMD) reduction [1], both at a local and generalised level. Bone damage is caused by an increased osteoclast activity and decreased osteoblast activation. This is mostly mediated by tumor necrosis factor (TNF)-*α*, interleukin (IL)-1, IL-6, IL-17, and receptor activator of nuclear factor kappa B ligand (RANKL) [2–5]. The erosion represents the final result of this process [6, 7] and it can be considered the central feature of bone damage of both RA and PsA, although in PsA there are significant differences when compared with RA, with a pattern characterised by concurrent erosions and new bone formation [8–10]. However, bone in the proximity of inflamed joints is susceptible to BMD reduction and it precedes erosive damage on X-ray [11–16].

Dual energy X-ray absorptiometry (DEXA, DXA) is the gold standard for measuring BMD [17]. Previous clinical studies demonstrated an association between hand BMD and RA severity, including disease activity, functional impairment and joint destruction [11–16, 18–22]. However, only a little data is available on the association between hand BMD reduction and disease activity in patients who are treated with disease modifying anti-rheumatic drugs (DMARDs) and TNF*α* inhibitors in a tight controlled setting [23–26].

The aim of this longitudinal observational study was to examine the BMD variations of the metacarpophalangeal joints (MCP) in patients affected by RA or PsA, in treatment with a DMARD-methotrexate-(MTX) or an anti-TNF*α* agent-etanercept.

## 2. Patients and Methods

All consecutive patients that attended our outpatients clinic for a six-month period (June–December 2011), affected by RA or PsA with hand joints involvement and with moderate or high disease activity as defined by the DAS28 (>3.2), were enrolled in this study. An RA diagnosis was made according to the previous ACR criteria [27], while a PsA diagnosis was made according to the Caspar criteria [28]. Patients could be either at the first diagnosis of disease or already in treatment with DMARDs but with a moderate-high disease activity as defined by DAS28 values, and they were eligible for anti-TNF*α* administration as defined by the Italian guidelines [29, 30]. Other inclusion criteria were an age of more than 18 years and a stable dose of steroids and methotrexate (for the second group) for the last 3 months. Exclusion criteria were pregnancy, other concomitant treatments that could influence BMD, malignancies, infectious diseases, chronic heart failure class III-IV according to the New York Heart Association (NYHA), severe pulmonary and hepatic diseases, unstable dosage of steroids or steroid doses superior of 10 mg of prednisone (or equivalent) for the second group of patients, or parenteral administration of steroids prior to the enrollment. A high dosage of steroids with quick tapering was allowed for the group at the first diagnosis, if administered for the first time. Nonsteroid anti-inflammatory drugs (NSAIDs) and local steroid injections in joints other than hands were permitted during the study. All patients agreed to participate in the study and signed an informed consent.

All patients underwent a clinical examination (all parameters necessary for the DAS28-CRP calculation) at the time of enrollment (time 0, T0) and after 1 month (T1), 3 months (T2), 6 months (T3), and 12 months (T4). At the time of enrollment, all patients also underwent an US examination of the MCP of both hands in order to assess the most “active” joint. All MCP were examined according to the EULAR recommendations [31], while inflammation was assessed using a semiquantitative score for synovial proliferation and power Doppler signal in a 0–3 scale as described previously [32]. The most active joint was the joint that reached the higher score for synovial proliferation plus a power Doppler signal. Joint effusion was not taken into account for this evaluation. Clinical examination and ultrasonography were performed by independent operators, blind to each others findings.

**Figure 1:**
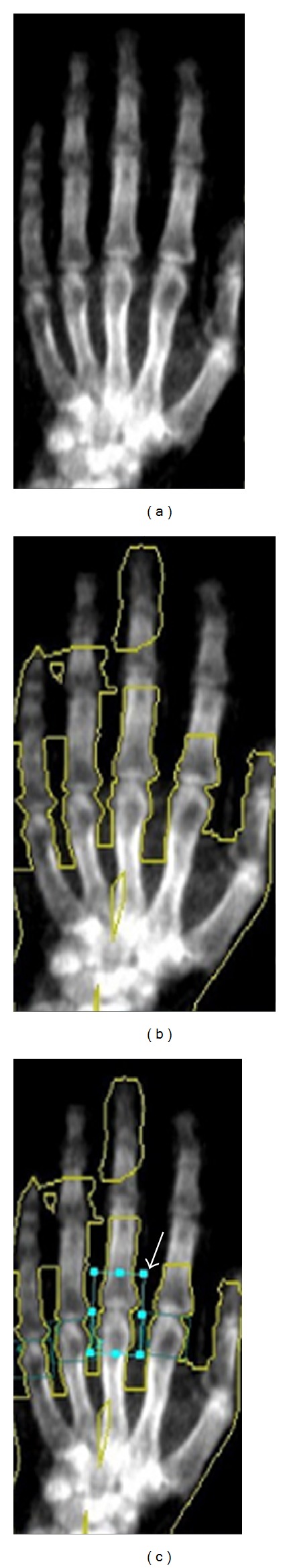
Acquisition and analysis of the MCP BMD at the first visit. The machine acquires the hand region (a) that has to be analysed manually. Then the operator defines the borders of the bone working in a magnified image with the software of the densitometer, obtaining a mask visible in the second image (white line) (b). Then he creates a ROI (region of interest, arrow) that includes the MCP rim, the head of the metacarpal bone, and the basis of the proximal phalange (c). Both the mask and the ROI are then saved and always used to assess BMD changes of each patient.

DXA examination of the hand, for the BMD assessment, was performed at T0, T2, T3, and T4. Joint BMD was measured at the most active joint, as defined at the US examination, with a dedicated region of interest (ROI) created ad hoc for the joint. Then the tool “compare mask” was used for the evaluation of the joint during the study in order to ensure the maximum reliability. In fact, the “compare mask” tool superimposes the images acquired during the followup and allows a very similar positioning of the ROI in the joint of interest (Figure 1). A Lunar Prodigy machine with the enCORE software was used for the study; the quality assurance data were collected daily to guaranty the performance of the scanners. The coefficient of variation (CV) of the machine used for the study has been previously tested for other sites and was never superior to 1.6% (lumbar spine 1.1%, femoral neck 1.5%, total femur 1.6%) [33]. Using the same machine, with a similar study design to ours (dedicated ROIs on MCP joints), Naumann et al. found a CV from 1.23% to 2.48% for MCP (MCP II–V: mean CV 1.16%; mean Least Significant Change 3.25%) [34].

Descriptive measures of demographic, disease-related, and DXA variables are presented as absolute and relative frequencies, mean and standard deviation (SD), or median and interquartile range (IQR) based on their type and distribution. The correlation between the variables has been calculated using multiple regression analysis as proposed by Bland and Altman [35]. Partial correlation coefficient between ΔBMD (beginning and end of the study) and the area under the curve of DAS28 over the study period has been used for the assessment of association between disease activity and BMD. Analyses were performed using STATA software package (StataCorp, 2009, release 11, TX, USA).

## 3. Results

Twenty-two patients (7 male, mean age of 49.9 years old, SD 12.4) were enrolled in the study. Of these patients 10 were affected by PsA and 12 by RA. Twelve patients were at the first diagnosis (early disease, 7 RA) and were treated with MTX and 10 patients (5 RA) were nonresponders to DMARDs and were treated with MTX plus an anti-TNF*α* agent (etanercept). The mean (SD) disease duration of the nonresponders was 29.9 (9.7) months. Demographic characteristics and baseline clinical data of the patients are summarised in Table 1.

**Figure 2:**
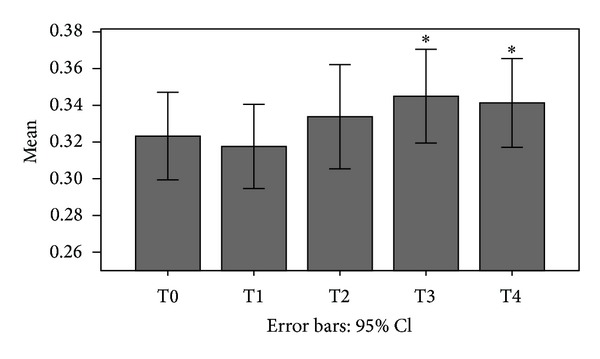
BMD variations of our cohort during the follow-up period. (*P < 0.01).

Globally, the mean BMD values increased during the follow-up period (Figure 2), in a statistically significant way, and also DAS28 improved. However, the BMD did not increase equally in all patient groups and in all treatment groups.

**Figure 3:**
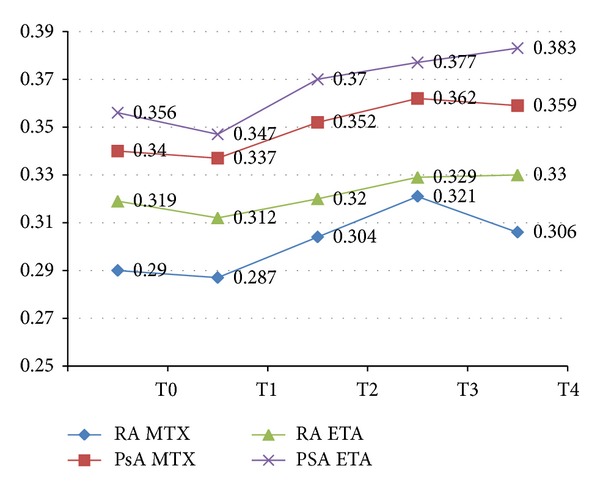
BMD values of the patients in the RA group and PsA group.

Patients affected by PsA presented with a higher BMD at the beginning of the study, despite the disease duration. In fact both new patients and nonresponders to MTX had a higher BMD (Figure 3). During the first month we assisted in a decrease of the BMD of the affected joint in all groups of patients. At the end of the study period patients in treatment with etanercept maintained the BMD increases obtained while patients in treatment with MTX had a slight reduction of the BMD at the last checkup, that resulted statistically significant only at the RA group (*P* < 0.05 versus T3).

**Figure 4:**
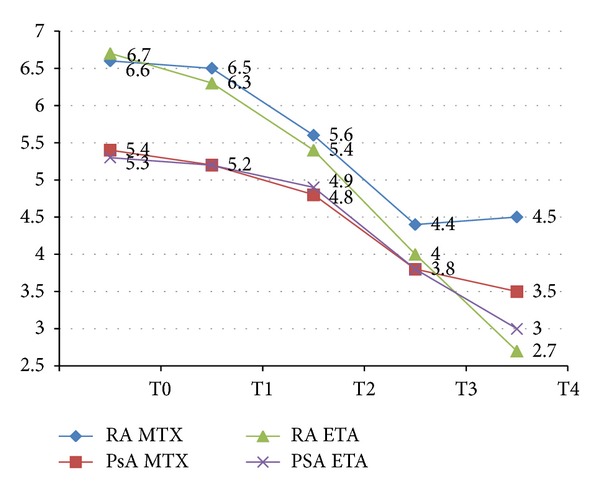
DAS28 values of the patients in the different groups during the study time.

DAS28 also improved in all patient groups. However in the MTX group we observed a higher score and not all patients reached a low disease activity with MTX alone (Figure 4).

Multiple regression analysis [35] was used to assess the correlation between disease activity and BMD of the MCP joints. The correlation coefficient is −0.56 with *P* < 0.0001. That means that the variation of DAS28 is associated with an inverse variation of the BMD of MCP joints. In other words, a decrease of the disease activity is associated with an increase of MCPs BMD and vice versa.

Further, the exposure at high disease activity scores is correlated with low values of MCPs' BMD independent of the sex or age of the patients (partial correlation coefficient −0.493, *P* = 0.028).

At 12 months, 5 patients were classified as “nonresponder” (3 affected by RA, in treatment with methotrexate, 2 affected by PsA, one in treatment with methotrexate and one in treatment with etanercept).

## 4. Discussion

Periarticular osteoporosis in patients affected by arthritis is a well-known phenomenon [1–5]. In fact, before the introduction of more sophisticated imaging techniques for the diagnosis of arthritis and joint effusion, the finding of MCPs transparency in traditional X-rays was one of the items of the 1987 ACR classification criteria for Rheumatoid Arthritis [27]. More recent studies have also demonstrated that periarticular bone loss occurs also in MCPs of patients affected by Psoriatic Arthritis [16, 23, 24] even if this aspect has been debated. In fact, periarticular osteoporosis is not considered by all authors as a marker of disease in the case of PsA, interpreting plain radiographs [8, 23, 24]. Using a BMD measurement, Reid et al. reported that BMD in 12 patients with PsA (assessed by metacarpal index) was not different from controls [36]. Whereas Cooper et al., using single photon absorptiometry (SPA) measurements of the distal forearm, noted bone loss in both RA and PsA, suggesting that their patients had more active disease than those of Reid et al. [37]. One previous study of our group assessed periarticular BMD in patients with various inflammatory arthropathies, and it found that periarticular bone loss occurred even in patients with PsA [38].

The pathogenesis of periarticular osteopenia in both rheumatoid and psoriatic arthritis is related to osteoclast activation by proinflammatory cytokines from the inflamed synovium, including prostaglandin E2, osteoclast activating factor, tumour necrosis factor (TNF), and interleukin 1 [1–5]. The ability of anti-inflammatory treatment to reduce hand bone loss in RA has been demonstrated in a double-blind study comparing oral prednisolone 7.5 mg/day for 2 years with placebo. The prednisolone group had less hand BMD loss, suggesting that the potent anti-inflammatory effect of prednisolone exceeded its negative effect on the bone [39].

With respect to the effects of anti-TNF therapy on hand bone loss in RA, only a few studies have been conducted. RA patients treated with anti-TNF therapy have been shown to have a lower rate of bone loss at the spine and hip than at the hand [18, 24–26]. In a 2-year longitudinal treatment strategy study (the BeST study), RA patients treated with anti-TNF therapy or high-dose prednisolone were shown to have a lower rate of bone loss at the hand than patients treated with conventional DMARD [18]. Furthermore, in a study employing quantitative ultrasound, the use of anti-TNF therapy had a positive effect on periarticular bone [40]. The beneficial effect of anti-TNF treatment on inflammatory-related hand bone loss in RA is supported by analysis from the PREMIER study, in which hand bone loss was assessed by digital X-ray radiogrammetry on the same hand radiographs scored with modified Sharp score [25]. In this study, the authors concluded that in patients with RA, potent anti-TNF therapy reduces the rate of inflammatory-related hand bone. This study also suggests that the bone damage disease process is still present in RA patients treated with TNF antagonists, even if radiographic joint damage on radiographs is apparently arrested and they suggest that quantitative measures of hand bone loss in RA patients can be recommended as outcomes for future clinical trials to detect ongoing bone damage.

In our series of patients, periarticular BMD increased after the third month of followup in both groups of patients except the last 3 months where we observed a negative trend in the group of patients affected with RA and treated with MTX. This could be due to the presence of 3 nonresponders in this group. This is in accordance with the previous observations on the effects of an appropriate anti-inflammatory therapy on bone loss in patients with RA [25, 39, 40]. In addition, we observed that BMD also increases in patients affected by PsA, independent of the treatment strategy. This observation is in accordance with the results of the study by Hoff et al. [23]. However, this is partially in contrast with the results of Szentpetery et al. [24]. In their study, they observed that there is a slight reduction of the periarticular BMD in MCPs either in patients affected by RA or PsA during the first year of treatment with an anti-TNF*α* agent. This is rather surprising and the same authors admitted that this result was rather unexpected.

In this study, the BMD variations have demonstrated to be inversely proportional to the disease activity defined by DAS28 scores. In previous studies, a good response to the treatment was correlated with reduced bone loss in patients affected by RA [25] and by PsA [23]. However, this is the first study where a BMD gain has been observed and not only with a reduction of the rate of bone loss. This could be due to the methods of the study. In our study we assessed only the most inflamed MCP joint according to clinical and US findings, so it is reasonable to expect a more dramatic response to the treatment either from the inflammation or from the bone damage point of view. In the other 2 studies, digital radiogrammetry (DXR) has been applied in more MCPs independently of the grade of inflammation of those joints. This could explain the less dramatic variation of BMD in these two studies. Also, in our study we discovered that there is a statistically significant correlation between persistence of disease activity and MCP BMD, independent of the age and sex of the patient.

Our study has some limitations that should be discussed. First of all the number of patients stratified by diagnosis, age, sex, and therapy was not sufficient to allow a statistical comparison between the various treatment groups, other than the descriptive ones. These analyses allowed us to observe that patients with a new diagnosis of RA tended to have a lower MCP BMD than patients already treated with a DMARD. Also PsA patients tended to have a higher BMD in respect to those affected by RA independent of the treatment administered. This is in accordance with previous observations [16]. Also, patients in combination therapy etanercept plus MTX, independent of the diagnosis, presented lower DAS28 scores and tended to obtain higher BMD values at the end of the followup in respect to the patients in monotherapy.

The assessment of only one, the most inflamed, MCP could be considered another limitation of our study. However, we chose to assess only one joint as in previous studies, the evaluation of more MCPs demonstrated a reduced rate or an arrest of bone loss [23, 25] while in our case we observed in some patients a gain of BMD at the end of the study period. It is reasonable to believe that the most inflamed joint reflects the bone damage caused by inflammation better. For example, in the study by Deodhar et al. [22], the authors assessed the bone mineral content of the whole hand and even if they also observed a reduced bone loss rate in the patients treated, the end of the study (5 years) resulted a consistent reduction of the BMC, especially during the first 3 years. We believe that the treatment options (only traditional DMARDs), the long study period, and maybe the inclusion of joints that are not typically affected by RA as well as the diaphysis of the hand's bones could have influenced the final results, adding the systemic bone damage (systemic osteoporosis) to bone damage due to local inflammation.

In conclusion, in patients affected by RA or PsA an effective treatment with either traditional DMARDs or anti-TNF*α* agents is effective in reducing periarticular bone loss in active MCPs. Bone loss reduction seems to be related to the treatment efficacy at a systemic level (reduction of DAS28 scores) more than the treatment agent itself even if patients treated with combination therapy tend to recuperate higher values of MCP BMD than those in monotherapy both in RA and PsA. Finally, further studies could be useful for assessing the role of the DXA measurements of affected MCPs and for defining the correlation between bone loss and erosive damage in RA and PsA.

## Figures and Tables

**Table 1:** Demographic and clinical data of the patients at baseline.

	Patients with RA		Patients with PsA	
	MTX group	Etanercept group	MTX group	Etanercept group
Patient, *N* (females)	7 (5)	5 (4)	5 (2)	5 (1)
Age, mean (SD)	45.7 (11.7)	60.0 (10)	45.4 (14.1)	50.2 (11)
Disease duration (months), mean (SD)	1.7 (0.2)	26.8 (9.5)	1.8 (0.3)	33 (9.5)
DAS28, mean (SD)	6.6 (0.9)	6.7 (0.9)	5.4 (0.7)	5.3 (0.6)
BMD of the examined joint, mean (SD)	0.291 (0.067)	0.319 (0.028)	0.340 (0.018)	0.363 (0.057)

PDUS: power Doppler US. DAS values between groups were not statistically significant.
